# Clarifying the Clinical Utility of NTAR/RGR for PAH and CTEPH. Comment on Iancu et al. Evaluating NT-proBNP-to-Albumin (NTAR) and RDW-to-eGFR (RGR) Ratios as Biomarkers for Predicting Hospitalization Duration and Mortality in Pulmonary Arterial Hypertension (PAH) and Chronic Thromboembolic Pulmonary Hypertension (CTEPH). *Diagnostics* 2025, *15*, 2126

**DOI:** 10.3390/diagnostics16010055

**Published:** 2025-12-23

**Authors:** Gianluca Pagnoni, Aurora Vicenzi, Francesca Coppi

**Affiliations:** 1 Cardiology Unit of Emergency Department, Guglielmo da Saliceto Hospital, 29121 Piacenza, Italy; dr.gianlucapagnoni@gmail.com; 2 National Institute for Cardiovascular Research (INRC), Via Irnerio 48, 40126 Bologna, Italy; 3 Department of Medical and Surgical Sciences for Children and Adults, University of Modena and Reggio Emilia, Via del Pozzo 71, 41124 Modena, Italy; francesca.coppi@unimore.it

We read with interest the work by Iancu et al., which introduces the composite NTAR and RGR indices for risk stratification in PAH and CTEPH and reports their performance for exceptionally long one-year overall survival (ELOS) and in-hospital mortality [1].

We find the contribution promising but in need of clarifications and methodological strengthening. The manuscript mentions multivariable logistic regression; however, the results presented appear largely unadjusted. The study would benefit from clearly specified models that include clinically relevant covariates (e.g., age, sex, WHO functional class, pulmonary hypertension, specific therapy, renal function, and comorbidities) to quantify the independent effect of NTAR/RGR. Because the unit of analysis is admissions, intra-patient dependence from repeat admissions should be addressed using mixed-effects models or generalized estimating equations (GEEs). The small number of in-hospital deaths warrants caution regarding stability and overfitting; bootstrap with optimism correction and the presentation of calibration metrics would be advisable.

It would also be useful to demonstrate incremental value over established markers such as NT-proBNP and albumin, addressing potential collinearity and employing net reclassification improvement (NRI), integrated discrimination improvement (IDI), and likelihood ratio tests. Sensitivity analyses should be provided to show how the NTAR/RGR ratios are constructed, including units/scales and standardization, to enhance interpretability. Given the managerial nature of the endpoints, a decision curve analysis would clarify the net clinical utility of integrating NTAR/RGR into care pathways, aligning with recent journal contributions that report on calibration, area under the curve (AUC) with confidence intervals, and decision curve analysis during external validation [2].

The framework aligns with recent evidence in CTEPH documenting the correlation between invasive measurements and echocardiographic estimates of right ventricular pressure and showing that NT-proBNP and right-heart functional parameters are associated with response to balloon pulmonary angioplasty (BPA) [3].

Independent observations in a separate systemic sclerosis cohort have shown that vitamin D insufficiency is associated with higher systolic pulmonary artery pressure (sPAP) and worse RV-PA coupling (lower TAPSE/sPAP), reinforcing the pathophysiological hypothesis that biomarkers and composite indices should be interpreted in light of hemodynamics and phenotype [4].

In parallel, in Sjögren’s syndrome, respiratory involvement shapes pulmonary hemodynamics early: in cases with interstitial lung disease, higher sPAP and reduced TAPSE/sPAP are observed, supporting echocardiographic monitoring and attention to RV-PA coupling in connective tissue diseases [5].

Looking ahead, we advocate prospective multicenter validation with follow-up, the pre-specification of minimal covariates and cut-offs, and integration with hemodynamic measurements and imaging to anchor biomarkers to mechanisms. In conclusion, NTAR and RGR are simple and potentially useful indicators, and with the proposed refinements, they could improve risk stratification and discharge planning in pulmonary hypertension.
